# Blood Drinkers

**Published:** 1874-11

**Authors:** 


Blood Drinkers.—Upon inquiry at slaughter-houses, it is found that there are nearly two hundred persons in the city of New York who are in the habit of drinking blood flowing warmly from oxen, for strengthening purposes and for the cure of certain diseases. A lady is reported to have spoken to an inquirer as follows: "Professor Velpeau, of Paris, prescribed blood for me. I was consumptive and hastening to the grave. It has prolonged my life fifteen years. I had the utmost repugnance for it at first, but now a half-pint of hot blood from a well-conditioned ox is the greatest luxury of my life. My sister's baby so far has been preserved and nourished with little else but blood. I know twenty persons who drink it in my own neighborhood, to whom I have recommended it. It has extraordinary effects on some people, especially women, but should not be resorted to unless there is absolute weakness of the system." On a visit of the inquirer to a slaughter-house in Tenth avenue, near Forty-second street, he found a delicate-looking woman with a sickly boy holding a glass to the blood which ran from an ox with his throat cut. Both drank two or three glasses in turn, and departed with an appearance of added vigor. One of the butchers was asked whether he ever drank blood, and is stated to have replied to the following effect: "Sure an' I do, now; why not, now? Faith, an' ye couldn't tell the difference between it an' milk. It's just as swate, shure, and in the winter it's warm and foine. Bedad, but it's stringthenin', sure. Hould on, and I'll get ye a dhrap. It's best warrum—runnin' right from the baste." The proprietor said, "All last winter we had men, women and children every morning to drink blood. They always imbibe beast's blood; never the blood of sheep. Some of them wince a bit at first, but, when you close your eyes, blood, warm from the beast's neck has just the same taste as warm milk from the cow. We don't charge for the blood, excepting when we sell it to sugar refiners." The blood of beeves is asserted to be more efficacious for weak lungs than cod-liver oil.—*The Laboratory.*

